# Ecological and subject-level drivers of interepidemic Rift Valley fever virus exposure in humans and livestock in Northern Kenya

**DOI:** 10.1038/s41598-023-42596-y

**Published:** 2023-09-15

**Authors:** Mathew Muturi, Athman Mwatondo, Ard M. Nijhof, James Akoko, Richard Nyamota, Anita Makori, Mutono Nyamai, Daniel Nthiwa, Lilian Wambua, Kristina Roesel, S. M. Thumbi, Bernard Bett

**Affiliations:** 1 Department of Veterinary Medicine, Dahlem Research School of Biomedical Sciences (DRS), Freie Universität Berlin, Berlin, Germany; 2 International Livestock Research Institute, Nairobi, Kenya; 3 Kenya Zoonotic Disease Unit, Ministry of Health and Ministry of Agriculture, Nairobi, Kenya; 4 Center for Epidemiological Modelling and Analysis–University of Nairobi, Nairobi, Kenya; 5 Department of Medical Microbiology and Immunology, University of Nairobi, Nairobi, Kenya; 6 Veterinary Centre for Resistance Research, Freie Universität Berlin, Berlin, Germany; 7 Institute for Parasitology and Tropical Veterinary Medicine, Freie Univesität Berlin, Berlin, Germany; 8 Paul G Allen School for Global Health, Washington State University, Pullman, WA USA; 9 Department of Biological Sciences, University of Embu, Embu, Kenya; 10 Institute for Immunology and Infection Research, University of Edinburgh, Edinburgh, Scotland, UK

**Keywords:** Microbiology, Ecology, Diseases, Risk factors

## Abstract

Nearly a century after the first reports of Rift Valley fever (RVF) were documented in Kenya, questions on the transmission dynamics of the disease remain. Specifically, data on viral maintenance in the quiescent years between epidemics is limited. We implemented a cross-sectional study in northern Kenya to determine the seroprevalence, risk factors, and ecological predictors of RVF in humans and livestock during an interepidemic period. Six hundred seventy-six human and 1,864 livestock samples were screened for anti-RVF Immunoglobulin G (IgG). Out of the 1,864 livestock samples tested for IgG, a subset of 1,103 samples was randomly selected for additional testing to detect the presence of anti-RVFV Immunoglobulin M (IgM). The anti-RVF virus (RVFV) IgG seropositivity in livestock and humans was 21.7% and 28.4%, respectively. RVFV IgM was detected in 0.4% of the livestock samples. Participation in the slaughter of livestock and age were positively associated with RVFV exposure in humans, while age was a significant factor in livestock. We detected significant interaction between rainfall and elevation's influence on livestock seropositivity, while in humans, elevation was negatively associated with RVF virus exposure. The linear increase of human and livestock exposure with age suggests an endemic transmission cycle, further corroborated by the detection of IgM antibodies in livestock.

## Introduction

Rift Valley fever (RVF), a zoonotic infection caused by a vector-borne *Phlebovirus*, is an important disease in Sub-Saharan Africa^1,2^. Cyclic, explosive outbreaks associated with significant morbidity in humans and livestock and restrictions on livestock trade are some of the primary reasons RVF is considered a regional priority^3–5^. Rift Valley fever virus (RVFV) was first identified in Kenya in 1931^6^. Since then, RVF outbreaks have been reported in the Arabian peninsula, North, West, and Southern Africa, and on islands off the East African coast, a testament to RVF's potential for intercontinental spread^7–11^.

In Eastern Africa, RVF occurs in two distinct ecological phases; an endemic phase lasting between five to ten years and an epidemic cycle lasting up to four months^12,13^. The epidemic phase is associated with above-average rainfall, usually linked to the El Niño-Southern Oscillation, which results in widespread flooding in low-lying areas^14–16^. Flooding and high ambient temperatures provide the requisite conditions for increased mosquito populations (the primary vectors), resulting in amplified disease transmission and consequent outbreaks in humans and animals^17–19^. Besides rainfall, other environmental factors that impact vector habitat suitability, such as soil type, vegetation cover, and elevation, may influence the magnitude of outbreak events during this phase^20,21^. The drivers of the epidemic phase are relatively well documented. However, there is comparatively less understanding of the viral maintenance dynamics during the quiescent years of the endemic phase. Two main hypotheses have been advanced on the same. The first is that RVFV is transmitted transovarially to *Aedes* mosquito eggs, which are laid in low-lying plains where they desiccate but remain viable for many years, explaining the long interepidemic periods (IEP)^22,23^. Flooding and warm temperatures provide ideal conditions for the eggs to rehydrate and hatch, giving rise to a large population of infected *Aedes* mosquitoes that transmit the RVFV to susceptible species, resulting in viral amplification and epidemics^24–26^. The second theory is based on the supposition that the virus is primarily maintained by a cryptic mosquito-host infection cycle that causes sub-clinical disease in susceptible livestock and wildlife species during IEP. The transition from the endemic phase to epidemic occurs when ecological conditions support a surge in the vector population and subsequent infection of a critical mass of naïve animals^27,28^. The latter hypothesis has gained credence over time due to increasing evidence of active infections in apparently healthy humans and livestock during IEP and the detection of RVFV antibodies in countries with no history of RVF outbreaks, such as the French Island of Mayotte, Chad, Tunisia, and Turkey^27–31^.

In Kenya, RVF outbreaks have been reported every five to ten years since the early 1930s^32^. However, there has been a marked increase in the frequency of detections in the last decade^33,34^. A few studies in the country provide evidence of IEP infections in humans and livestock; however, questions around the primary reservoirs, effects of herd immunity on RVF emergence, species susceptibility, and ecological drivers of RVFV infection during IEP still persist^35–39^. Routine sero-monitoring is vital in answering these questions and generating valid data for risk assessments^40^. Furthermore, the importance of prevalence data in complementing climatic and ecological parameters to improve the spatial specificity of RVF prediction models is well described in literature^21,41^. Our study sought to establish the IEP prevalence of RVF, and factors associated with RVFV exposure in Isiolo County, an RVF epidemic-prone region, through a livestock-human-linked study to generate empirical data to guide early detection and response.

## Materials and methods

### Ethics statement

Ethical approval for the study was provided by the International Livestock Research Institute Institutional Research Ethics Committee (Reference number: ILRI-IREC2020-07) to sample livestock and humans. Livestock owners and household heads provided written consent before sampling. For individuals under 13 years, written permission was obtained from their parents or legal guardians. For those between the ages of 13 and 17, written assent and written parental/legal guardian permission were obtained. Adults over the age of 18 provided written consent before sampling. The human and animal, sampling, and data collection were performed in accordance with the standard operating procedures and guidelines outlined in the ethical approval.

### Study area

**Figure 1:**
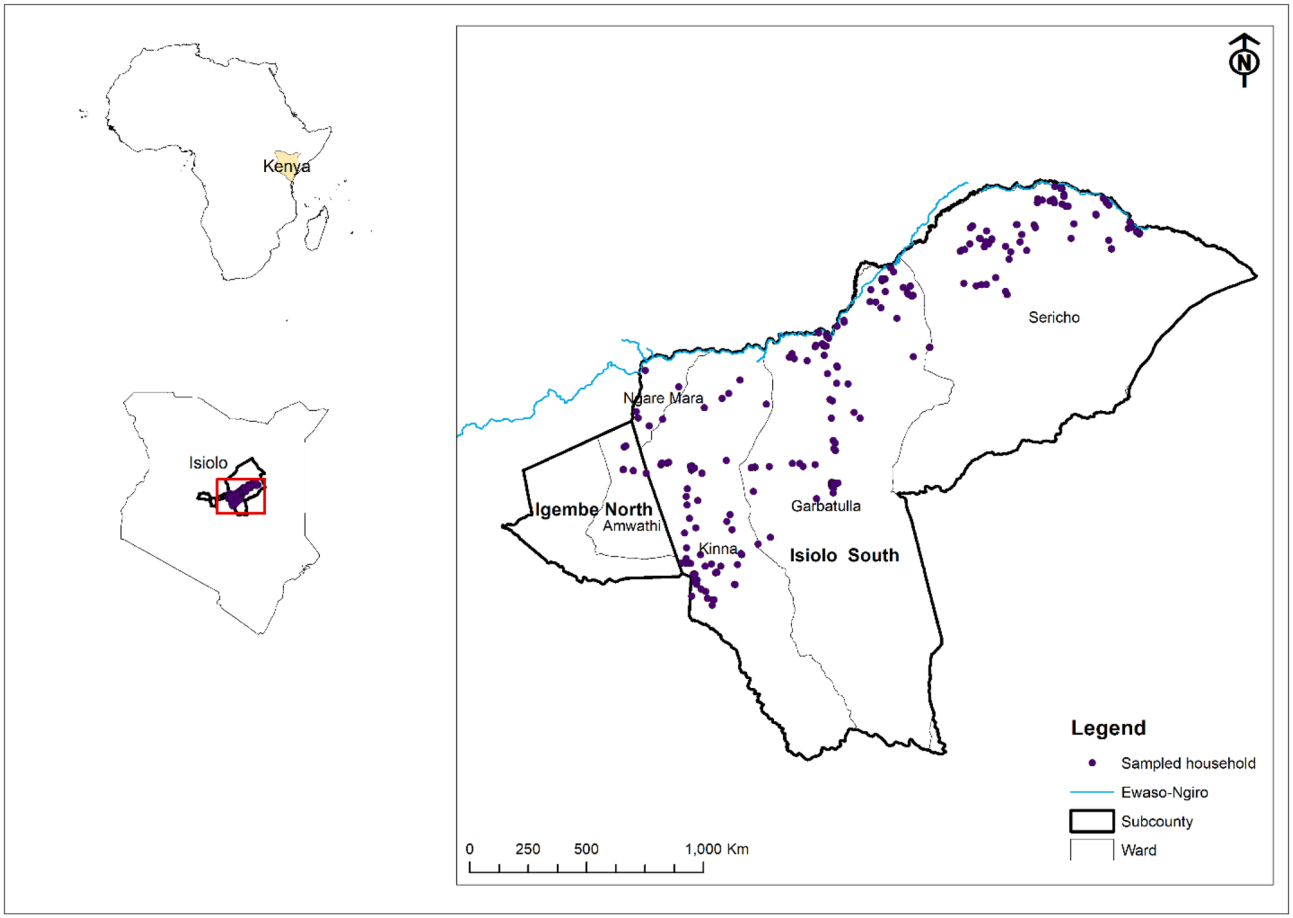
Map of Africa showing the location of Kenya, the study site in green (Isiolo County), and specific wards and distribution of randomly sampled households. Shapefile used to create the map was obtained from: https://gadm.org/download_country.html.

We conducted the study in three wards (administrative sub-units) in Isiolo South: Sericho, Kinna, and Garbatula (Fig. 1). Isiolo County was selected for the study because the last RVF outbreak in Kenya (November 2020 to January 2021) primarily affected the county, although Isiolo is considered a medium-risk area^42^. The area is a low-lying region with a mean annual rainfall of 300 to 350 mm distributed between two wet seasons: March to April and October to December^43^. Precipitation is usually erratic, and droughts are frequent. Although the primary economic activity is nomadic pastoralism, there are limited farming activities on the flood plains of the Ewaso-Ngiro, which is the only permanent river in the region^43^. The main livestock species in the area are cattle, camels, sheep, and goats, with the latter having the highest population. In addition to livestock, the area has a high population of free-roaming wildlife herbivores: African buffalo (*Syncerus caffer*), plains zebra (*Equus quagga*), and waterbucks (*Kobus ellipsiprymnus*) which provides an opportunity to study the epidemiology of zoonotic pathogens at the human-livestock-wildlife interphase. The Borana people, around 100,000, are sparsely distributed across the study area, which covers approximately 9,902 km^2^^44^. The mean elevation of the study area ranges from 282 m in Sericho to 459 m in Garbatulla to a high of 589 m in Kinna.

## Study design and sample size determination

The study was a household-based cross-sectional survey of humans and livestock (sheep, goats, and camels), conducted between July and August 2021. Cattle were not sampled because there was a vaccination campaign targeting cattle in part of the study area, six months before the study. The sample sizes for both livestock and humans were determined using the formula; $n=\frac{{z}^{2}P\left(1-P\right)}{{d}^{2}}$ where the $z$ is the standard normal distribution value corresponding to a 95% confidence interval, $P$ is the expected prevalence, $d$ is the precision level set at 0.05, and $n$ is the minimum sample size^45^. A priori prevalence of 50% was used to estimate a sample size of 385 in humans and livestock because current exposure estimates in the study area were unavailable. The minimum sample size in humans and livestock was multiplied by a design effect to account for variance in precision because of multistage sampling. The design effect (Deff) was estimated using the formula; $Deff=1+(b-1)\rho$ where $b$ was the number of projected samples per herd/household and $\rho$ is the intra-cluster correlation coefficient (ICC), which is the measure of the rate of homogeneity. In both humans and livestock, a value of 0.2 was used for ICC based on evidence that the upper estimates for this parameter do not usually exceed 0.2 except for highly infectious viral diseases^46,47^. Based on the fact that average herd sizes are highly variable in pastoral set-ups and we had limited data on herd sizes and composition, in the study area, we used 20 animals as the cluster size (b); this is based on a similar study done in a comparable pastoral setting in a neighboring county to our study area^48^. This gave a design effect of 4.8 and an adjusted sample size of 1,843 for livestock. In the human sample size estimation, we used an average cluster size of three, deduced from a similar study giving a design effect of 1.4 and a minimum sample size of 538 humans^48^.

### Sampling design and data collection

The study was designed as a linked human-livestock study where livestock samples were collected from households from which humans were sampled. Two-stage sampling was used, with households and animals/human subjects being the primary and secondary sampling unit, respectively. A map of the study area was developed in Arc-GIS, and 250 random geographical coordinates (including additional points for replacement) were generated to identify households for sampling. A household closest to a coordinate was selected for inclusion. Inclusion criteria for livestock sampling were herds where at least five clinically healthy animals of one of three study species (camels, sheep, and goats) species were available for sampling, herds with no history of RVF vaccination, and livestock owned by households that consent to human sampling. Information on livestock species distribution in herds in the study area was unavailable, so a maximum of five animals of each available species were sampled per herd using systematic random sampling. For systematic random sampling, we calculated the sampling interval using the formula $i=N/n$  where $i$ was the sampling interval, $N$ was the herd size, and $n$ was five. A trained veterinarian collected up to 10 mL of blood from each animal using jugular venipuncture.

All consenting humans, excluding children under two years old, were enrolled in households with or without livestock. A nurse collected five mL of blood using non-heparinized vacutainer tubes. Human and livestock blood samples were transported to a field laboratory using cool boxes that maintained a temperature of 4 °C to 8 °C for processing. Sera were collected through centrifugation at 2500 g for 15 min and transferred into barcoded cryovial tubes, which were stored and transported at −20 °C to the International Livestock Research Institute (ILRI), Nairobi, using a motorized freezer.

Data on potential risk factors for RVFV infection, like demographic information in livestock and humans, livestock husbandry practices, livestock reproductive losses (including cattle), and human contact with livestock, were collected and managed electronically through structured questionnaires in Open Data Kit (ODK) database systems version 1.30.1.

### Laboratory testing

Serological analysis of human and livestock sera samples for anti-RVFV Immunoglobulin G (IgG) antibodies was done using a multi-species competitive enzyme-linked immunosorbent assay (cELISA) from ID.vet (Innovative Diagnostics, Grabels, France). Livestock testing for anti-RVFV Immunoglobulin M (IgM) antibodies was done using IgM antibody capture ELISA for the detection of anti-nucleoprotein IgM antibodies in ruminant serum and plasma from ID.vet. These kits were selected due to their reported high sensitivity and specificity values^49^. A total of 1,864 livestock samples and 676 human samples were tested using the anti-RVFV Immunoglobulin G (IgG). A subset of the livestock samples, 1,103 randomly selected from the 1,864 samples tested for IgG, were additionally tested for anti-RVFV Immunoglobulin M (IgM). Human samples were not tested for IgM because, unlike the IgG kit, the IgM kit is only validated for use in ruminants. All testing was conducted as per the manufacturer's instructions. Briefly, 100 µl of diluted serum samples and kit positive and negative controls were loaded onto precoated microtiter plates and incubated at 37 °C for 1 h. The plates were then washed three times using 1X wash solution followed by adding 100 µL 1X conjugate solution. The reaction was incubated at room temperature for 30 min and then washed thrice. The substrate solution (100 µL) was added to each well, and the test plate was incubated at room temperature for 15 min. The reaction was stopped by adding 100 µL of the stop solution, and the optical densities (O.D.s) were read at 450 nm wavelength using a BioTek ELISA reader (Synergy HT, United States). The sample competition percent (S/N%) was determined by dividing the mean O.D. of each sample by the mean O.D. for the negative control and multiplying by 100%. An animal was considered positive if S/N% was ≤ 40%, negative if > 50%, and borderline if the value was between 40 and 50% as per the manufacturer's recommended cut-off values. Validation of the test results was done by duplicate testing of all assays. Serological analysis for all samples was done at the ILRI in Nairobi.

### Data analysis methods

Data analysis was done using R statistical environment, version 4.1.2^50^. After data merging and cleaning, initial descriptive statistical analyses included the estimation of the overall seroprevalence of RVF with 95% confidence intervals (CI) in livestock and humans at the animal/individual and herd/household levels using RVF IgG data (we defined a seropositive household or herd as any household with at least one seropositive human or animal, respectively) generated through cross-tabulations. These results were generated through cross-tabulations using the *propCI* function in the *prevalence* package. Prevalence was tabulated for each categorical variable. In livestock, the categorical variables considered included animal sex (male and female), species (camels, goats, and sheep), and age (less than one year and above one year). The above descriptive statistical analyses were further calculated for each livestock species. Human seroprevalence estimates were also stratified by demographic characteristics.

Household size was categorized into two levels (≤ five and > five household members), while herd size was categorized into three levels (≤ 100, 101–200, 201–300, and > 300 animals). The Chi-square (χ^2^) was performed to determine if differences between variables were significant and to assess the relationships between categorical variables and seropositivity of RVF IgG in livestock and humans. Positive samples by IgM ELISA were classified as recent exposures because these antibodies are known to last between four to 72 days post-exposure and are usually undetectable six months post-exposure^51,52^. Samples that remained borderline after retesting were removed from the analysis.

### Statistical modeling

Two models were used, one for human and another for animal-level data, with the level of IgG RVF exposure at the human subject/animal level being used as the outcome variable in both models. We used a hierarchical Bayesian modeling algorithm that applies both Integrated Nested Laplace approximation (INLA) to account for the clustering of data and stochastic partial differential equation (SPDE) to estimate model parameters and account for spatial effects. These models were fitted using the R-INLA function in R (R-INLA version 4.1.1.)^53^. A hierarchical model was preferred to the standard analysis based on evidence that showed RVFV exposure is influenced to a large extent by spatial variability^54,55^. A mesh was created to help capture relative locations of the sampling points and herds (Supplementary Figure S1).

For both models, subject-level, herd, and ecological-level variables were used as predictors in the model. The animal-level variables considered were age, sex, and species. For humans, the variables considered were sex, age, and risk practices. The herd level variable was herd size. The ecological predictor variables for RVF transmission were selected based on their possible role in RVF transmission, as described in the literature. These included soil type, slope, elevation, soil texture, distance to a river, normalized difference vegetation index (NDVI), land surface temperature(LST), land use, and land cover^20,55–57^. Table 1 details predictors of RVFV transmission and their sources.

**Table 1:** Predictors of RVFV transmission and their sources.

Dataset	Units	Source	Spatial resolution
Soil type		ICPAC geoportal (https://geoportal.icpac.net/layers/geonode%3Asoils)	
Soil texture		ICPAC geoportal (https://geoportal.icpac.net/layers/geonode%3Asoils)	
Distance to river	Meters	ICPAC geoportal (https://geoportal.icpac.net/layers/geonode%3Asoils)	
NDVI*		Google earth engine	30 m
LST*	°C	Google earth engine	30 m
Land use/land cover		Google earth engine	30 m
Rainfall*	Millimeters (mL)	https://data.chc.ucsb.edu/products/CHIRPS-2.0/africa_3-monthly/tifs/)	5 km
Elevation	Meters	https://www.diva-gis.org/gdata)	30 m

*NDVI, LST, and rainfall data are for the period between 15th July and 17th August.

### Model building

For both models, progressive forward and backward variable selection procedures were used to identify and select fixed factors that could be retained in the model. For the first model, a variable or interaction terms were retained in the model if their inclusion minimized the Deviance Information Criterion (DIC). For continuous variables, the linearity assumption was evaluated by fitting and evaluating the significance of their quadratic terms. The significance of the spatial effect in both models was tested using DIC statistics.

## Results

We collected samples from 676 humans in 237 households and 1,864 livestock sera samples (96 camels, 828 sheep, and 940 goats) from 201 households. The median age of the humans sampled was 37.1 years old (range 4–82). The majority of the study participants were male (83%). On average, we sampled three humans per household (range 1–8), and the median household size was four (1–15). Most study participants had no formal education (75%); less than 10% had completed high school education. The median herd size of all livestock combined was 155 (ranging from 8 to 1,113), while the median herd/flock sizes for camels, goats, and sheep were 40 (range: 19–100), 79 (range: 8–546), and 73 (range: 4–626), respectively.

Of the total number of livestock-keeping households surveyed, 62% owned two different types of livestock, 21.16% owned only one species, and only 15% owned three different types of livestock. The median number of animals sampled in each household was 10 (range: 1–15). Most animals sampled were female (77%), and 86% of livestock samples were from animals above one year (Table 2).

**Table 2:** Livestock (A) and human (B) level risk factors assessed for their association with IgG RVF seropositivity in Isiolo County.

(A) Livestock				
Variable	Category	n (1864)	% Seropositivity (95% CI)	${X}^{2}$;$P>Z$
Species	Camels	96	13.5% (7.4–22.0%)	4.9; 0.08
–	Goats	940	21.3% (18.7–24.0%)	
–	Sheep	828	23.2% (20.4–26.2%)	
Sex	Female	1428	23.4% (21.2–25.7%)	9.5; **0.002**
–	Male	436	16.3% (12.9–20.1%)	
Ward	Kina	525	22.5% (19.0–26.3%)	18.3; < **0.001**
–	Garbatulla	935	18.3% (15.9–20.9%)	
–	Sericho	404	28.7% (24.3–33.3%)	
Age	Young (1–6 months)	38	7.9 (2.6–16.8)	19.7; < 0.001
	Weaners (6–12 months)	226	11.9 (8.4–16.3)	
	Adults (> 12 months)	1600	23.4 (21.4–25.6)	
Herd size	< = 100	570	21.2% (17.9–24.8%)	4.5; 0.2
–	101–200	506	24.7% (21.0–28.7%)	
–	201–300	391	18.9% (15.2–23.2%)	
–	> 300	397	21.4% (17.5–25.8%)	

(B) Humans				
Variable	Category	n (676)	% Seropositivity (95% CI)	${X}^{2}$;$P>Z$
Sex	Female	116	23.3% (15.9–32.0%	1.5; 0.2
–	Male	560	29.5% (25.7–33.4%)	
Ward	Kina	178	19.7% (14.1–26.3%	18.3; < **0.001**
–	Garbatulla	264	25.8% (20.6–31.5%)	
–	Sericho	234	38.0% (31.8–44.6%)	
Age	< 18 years	91	8.8 (4.4–14.7)	48.61; < 0.001
	18–40 years	330	22.6 (18.4–27.3)	
	> 40 years	255	42.7 (36.9–49.3)	
Education	Have formal education	170	20.0% (14.3–26.8%)	7.3; **0.007**
–	No formal education	506	31.2% (27.2–35.5%)	

Significant values are in bold.

We evaluated the prevalence of livestock abortion in the three months preceding the study as it is considered one of the primary syndromes indicative of RVFV infection. We used adult female animals as the denominator in our calculations; 131 cattle, 60 camels, 570 sheep, and 655 goats. The prevalence of abortion was observed in goats at 37.1% (95% CI 33.4–40.9), followed by camels at 15% (95% CI 7.1–26.6%), and cattle at 19.8% (95% CI 13.4–27.7). Sheep had the lowest reported abortion rate at 10.9% (95% CI 8.4–13.7). Overall, the herd-level prevalence of abortion in the three months preceding our study was 85.9%.

### Livestock Rift Valley fever virus seroprevalence by cELISA

Overall, livestock anti-RVFV IgG seropositivity was 21.7% (95% CI 19.9–23.7%), and the livestock household positivity (households with at least one seropositive animal) was 64.2% (95% CI 58.9–71.4). Among livestock, the prevalence was highest in goats (Table 2). RVF seropositivity in animals significantly differed by age, animal sex, and location (Table 2).

### Livestock Rift Valley fever virus seroprevalence by IgM ELISA

Four of 1,103 randomly selected livestock samples (535 sheep, 471 goats, 97 camels) tested positive using anti-RVFV IgM antibody Capture (MAC) ELISA giving an overall positivity rate of 0.4% (95% CI 0.1–0.9%). All the IgM-positive livestock were sheep; of these, only one was positive for both IgG and IgM.

### Human Rift Valley fever virus seroprevalence by cELISA

The individual human seroprevalence was 28.4% (95% CI 24.9–31.90), while the human household seroprevalence was 53.5% (95% CI 46.60–59.65). Although females had a lower seropositivity of 23.3% compared to males at 29.5%, the difference was not statistically significant. Age, location, and education level were, however, significant (Table 2).

### Multivariable analysis

**Figure 2:**
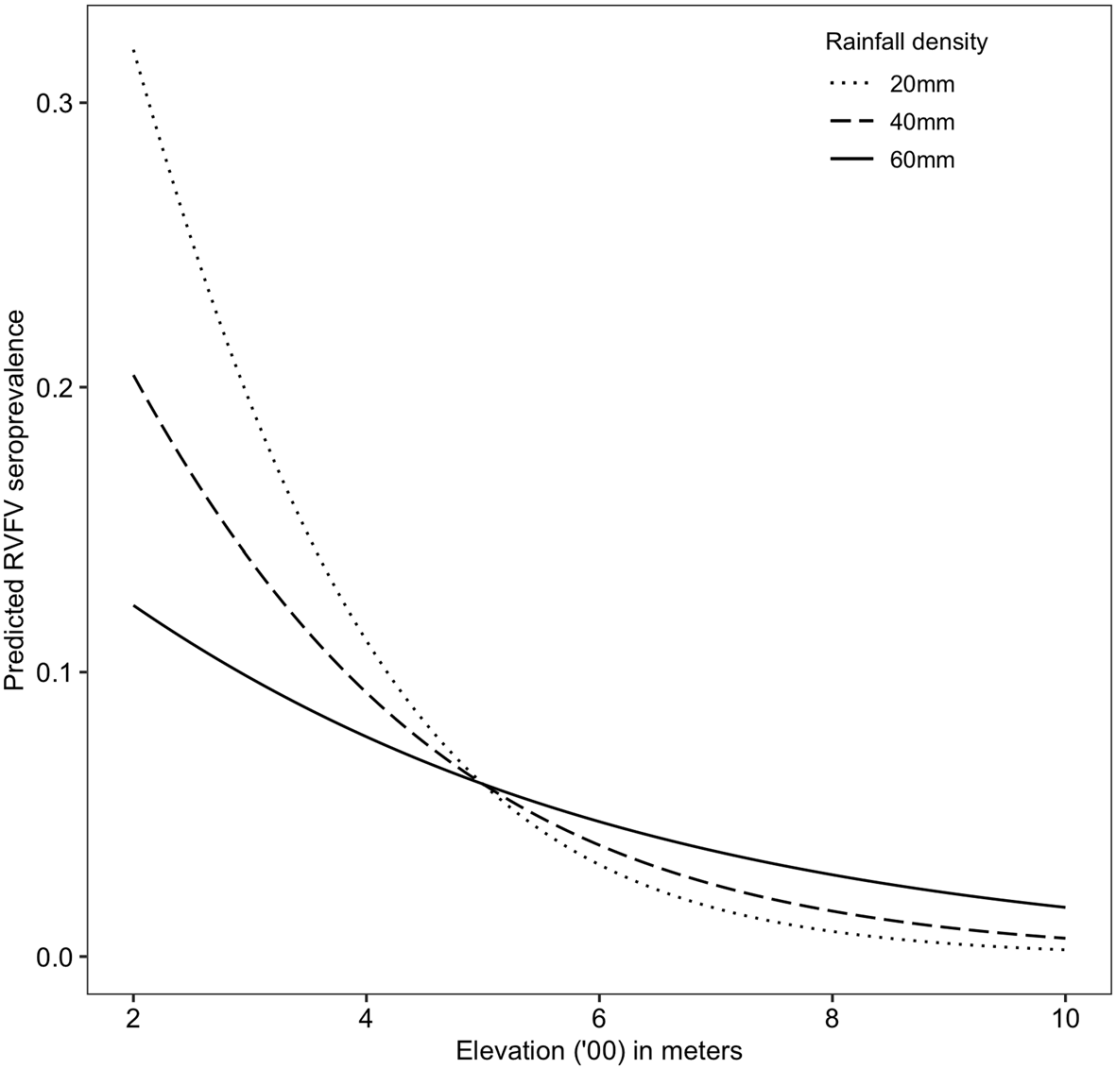
Interaction between rainfall and elevation in influencing RVFV exposure in livestock.

Results from the multivariable analysis of animal and human data are shown in Tables 3 and 4, respectively. The animal-level factors that were significantly associated with RVFV seroprevalence included sex and age. Female livestock had significantly lower odds of RVFV infection compared to males. Younger livestock, including suckling animals and weaners, had significantly lower odds of RVF exposure compared to adults (Table 3). Out of the eight ecological variables, elevation was found to negatively influence RVF exposure in livestock while rainfall positively influences RVF exposure. However, further analysis reveals significant interaction between the two variables because the rate of decline of RVFV exposure with elevation was faster in areas with lower rainfall than in areas with higher rainfall (Fig. 2). The presence of a significant interaction between mean rainfall and elevation implies that the effects of these two variables on RVFV seroprevalence are interdependent and should not be evaluated in isolation. Household herd sizes were not associated with RVF exposure in livestock and were therefore removed from the final multivariable model. DIC estimates of models with and without the spatial random effect were 2028.23 and 2193.27, respectively, indicating a significant spatial autocorrelation in the data.

**Table 3:** Association between animal-level and ecological factors and RVF IgG seropositivity in Isiolo County.

	Odds ratio	2.5% quantile	97.5% quantile
Sex			
Male	Ref	1	1
Female	0.62	0.45	0.84
Age			
Young (1–6 months)	0.19	0.04	0.66
Weaners (6–12 months)	0.45	0.28	0.71
Adult (Above 12 months)	Ref	1	1
Species			
Camel	0.87	0.39	1.85
Sheep	1.11	0.87	1.42
Goat	Ref	1	1
Ecological predictors			
Elevation	0.44	0.26	0.69
Mean annual rainfall	0.96	0.89	1.02
(Elevation/100) * mean rainfall)	1.01	1.00	1.02

*Ref* Reference category.

*Elevation divided by 100 to obtain stable parameter estimates.

**Table 4:** Association between human variables and RVF seropositivity in Isiolo County.

Variable	Level	Odds ratio	2.5% quantile	97.5% quantile
Age (years)	< 18	0.37	0.18	0.71
	18–40	Ref	1	1
	> 40	0.98	0.6	1.36
Participate in animal slaughter	No	0.57	0.35	0.90
	Yes	Ref	1	1
Elevation		0.79	0.71	0.89

### Human data

In humans, age, participation in animal slaughter, and elevation were found to be significantly associated with RVFV exposure (Table 4). Younger people (< 18 years) had lower odds of RVFV exposure compared to middle-aged individuals (18–40 years). Participation in the slaughter of animals was associated with higher odds of exposure. Similarly, people who lived in lower altitude areas had higher odds of exposure compared to those who lived in elevated areas. DIC estimates of models with and without the spatial random effect were 736.61 and 737.47, respectively, indicating that the spatial component substantially improves the fit of the model.

## Discussion

This study provides evidence of RVFV exposure in humans and livestock and RVFV circulation during the IEP period. We also provide data quantifying the influence of spatial variability in RVFV infections in a defined ecosystem that has had repeated outbreaks of the disease. The detection of RVFV IgM antibodies in non-clinical study animals provides evidence of recent exposure. This supports the hypothesis that RVF could be primarily maintained through cryptic transmission in-between outbreaks because these antibodies are known to persist for four days to two months post-exposure in sheep while in cattle, the highest mean level of IgM detection is within the first 30 days although antibodies could be detected in a small proportion of cattle (3.6%) up to five months post-exposure^51,52^. Similar studies have also reported low rates of subclinical, active infection during IEP in Kenya and Tanzania, not only building evidence of the role of cryptic transmission in RVF maintenance but also reaffirming the challenges of RVF passive surveillance during non-outbreak periods^39,58^. An interesting data point from our study was that all the IgM-positive animals were sheep. Although we cannot explicitly infer this to mean sheep are more susceptible to RVFV exposure during IEP, multiple other studies have reported the same finding^59–62^.

The high IgG RVF prevalence in livestock was not unexpected; Isiolo has had a history of RVF outbreaks (the last outbreak reported was in December 2021), and the area has suitable habitat for RVFV vector survival^20,32,63^. Transhumance pastoralism, the primary livestock production system practiced in the region, could also contribute to the high exposure rates because livestock movement is a primary pathway for the introduction of RVFV into susceptible populations^64,65^. Interspecies differences between goats, sheep, and camels were not significant, which we postulate to be a factor of similarities in exposure for animals living in the same environment. We found no published data on the IEP prevalence of RVF in small ruminants in Isiolo. Our results are, however, comparable to livestock prevalence of 25% and 28% reported in the neighboring counties of Tana-River and Garissa, respectively, providing further insight into the endemicity of RVF in northeastern Kenya^66,67^. Our data on RVF exposure in camels makes for an interesting discussion point because studies providing evidence of camel exposure to RVFV in Kenya are very few and far between. We found a seroprevalence of 14% in camels, the same as we reported in a 2020 study^68^, corroborating previous assertions that camels could play a significant role in RVF maintenance. Other studies in East Africa have reported varying seroprevalences, 28% in Tanzania^69^ and 43% in Ethiopia^70^, further justifying the urgent need to invest in RVF active surveillance studies in Kenyan camels.

Surveillance for livestock abortion is essential in RVF detection and response because abortions are one of the earliest observable signs of RVF^12^. As such, establishing baseline abortion levels in livestock is critical in determining thresholds for event-based RVF early warning systems^34^. However, there is very limited data on abortion surveillance in Kenya, and we believe this is the first report on the prevalence of abortion in pastoral herds in Kenya. A recent study in Tanzania, however, reported comparable results; the prevalence of abortion was highest in goats, followed by cattle, and least in sheep. The Tanzanian study detected an outbreak of RVF during an interepidemic period, exemplifying the utility of abortion surveillance as a tool in RVF detection^71^.

The significant association between livestock seropositivity and age, a finding commonly reported by similar studies in and outside Kenya, implies continuous viral exposure over time, a defining characteristic of enzootic transmission cycles^66,67,72,73^. Our data shows female livestock had lower odds of exposure. Literature on the association between sex and RVF positivity in livestock is contradictory; several studies report higher odds of infection in females^66,72,74,75^, some report no association^58,73,76^, and others, like ours, find higher odds of exposure in males^77^. The varying statistics indicate that it is improbable that sex has a significant association with RVF positivity. The differences could be attributed to differences in study sites and techniques, or as we hypothesize in our study, differences in livestock management practices such as animal movement and vector control between male animals and the more valued female livestock.

As is the case in livestock, the high individual and household prevalence in humans indicates the endemicity of RVF. A survey done in the same study area six years prior reported a human prevalence of 26.7%, closely mirroring our 24% finding, reaffirming the almost hyperendemic status of RVF in Isiolo^78^. Other studies in Kenya and Tanzania have reported widely varying positivity ranges, from 4.5% in Serengeti to 39% in Kenya, which could be primarily a factor of spatial variation or risk practices^78,79^. We attribute the high human seroprevalence to the pastoral lifestyle, characterized by regular contact with potentially-infected livestock and livestock products and the abundance of potential RVFV vectors in the study area^63,78,80,81^.

Risk factor analysis revealed slaughtering and butchering animals and age was associated with higher odds of RVFV exposure. Direct human contact with infected animal tissues has long been reported to be one of the main pathways for zoonotic transmission of RVFV, explaining why slaughtering and butchering is a common risk factor in many studies^78,82,83^. This underlines the importance of awareness creation on infection prevention control practices among high-risk groups like pastoralists, meat handlers, butchers, and slaughterhouse workers. We found age has a positive effect on seropositivity; this demonstrates continuous viral exposure over time. The fact that older populations are more likely to be involved in risky practices like slaughtering and butchering animals could also contribute to the age-dependent variance^66,78^. In our study, there was no association between sex and RVFV seropositivity. Reports on the association between sex and human exposure are heterogeneous. Some studies report males and females have different odds of infection due to cultural-gender roles predisposing them to distinct high-risk practices^83^. In contrast, others do not report significant associations. In our study, we did not find a significant association between sex and RVFV exposure, a finding reported by another RVF cross-sectional study in Kenya^37^. We postulate this could be due to similarities in viral exposure between males and females in our study area.

Our analysis shows humans and livestock in Sericho ward had significantly higher RVFV seropositivity, leading us to investigate the possible role of ecological variability on RVFV exposure in humans and livestock. We found a significant interaction between rainfall and elevation in their influence on livestock seropositivity meaning areas with low rainfall densities (e.g., 20 mm/year) and low altitude ~ 200 m above sea level will generally have high baseline RVFV seroprevalences. These areas are likely to be flood plains in areas like Sericho ward which often suffer high infection pressures during the wet season. It would therefore be expected that the prevalence of the disease would drop considerably with increasing altitude because, at higher altitudes, incidences of flooding will be minimal. Conversely, areas with higher rainfall levels (e.g., 60 mm) would be able to sustain RVFV infections regardless of whether they are in lower or higher altitudes. This is because the seasonal rains received would be able to support mosquito populations. Elevation influences the amount of precipitation, possibly explaining the correlation between the two variables. Additionally, rainfall provides water pools required for mosquito vector larvae development and eventual survival in the dry season, which could explain the significant linkage between elevation, rainfall, and seropositivity^25,57^. Elevation was a significant predictor variable for seropositivity in humans. The impact of elevation alone on RVF can vary based on local ecological and climatic conditions, but lower elevation areas below 1000 m are generally warmer and permissive of vector survival compared to high altitude areas explaining the inverse relationship between RVFV seropositivity and elevation^10,27^. Other ecological determinants like soil type investigated were not significant, which could be a factor of their homogeneity in our small study area compared to studies done over vast regions.

There were several limitations to our study. One was insecurity in parts of our study area, making it impossible to sample the entire target area. The other limitation was we did not perform a confirmatory plaque reduction neutralization test to rule out false positives and cross-immunoreactivity with viruses in the same family. An inherent weakness of crossectional studies is that study participants are expected to recall and report exposures subject to bias. The results suggest cryptic transmission of RVFV with spatial variability in exposure driven by elevation and rainfall in northern Kenya. Our data did illustrate the association of demographic and behavioral risk practices to RVFV, which can be used to define most at-risk groups. The data demonstrates the need for more extensive studies to elucidate further the true burden of RVF and the drivers of infection in high-risk regions. Our findings demonstrate the need for multi-species, prospective, active surveillance studies to better understand transmission dynamics during IEP. This data will guide the design of effective interventions to minimize future outbreaks' impact on vulnerable populations.

### Supplementary Information

Supplementary Figure S1.

## Data Availability

All data is completely available without any restrictions. Please contact the corresponding author at muturimathew@gmail.com for any data requests.
