# Supplementary material for: Ecological and subject-level drivers of interepidemic Rift Valley fever virus exposure in humans and livestock in Northern Kenya
Source: Sci Rep. 2023 Sep 15;13:15342. doi: 10.1038/s41598-023-42596-y (PMC10504342; doi:10.1038/s41598-023-42596-y)
Supplement: Supplementary file 1 — Supplementary Figure S1. [file 41598_2023_42596_MOESM1_ESM.docx]

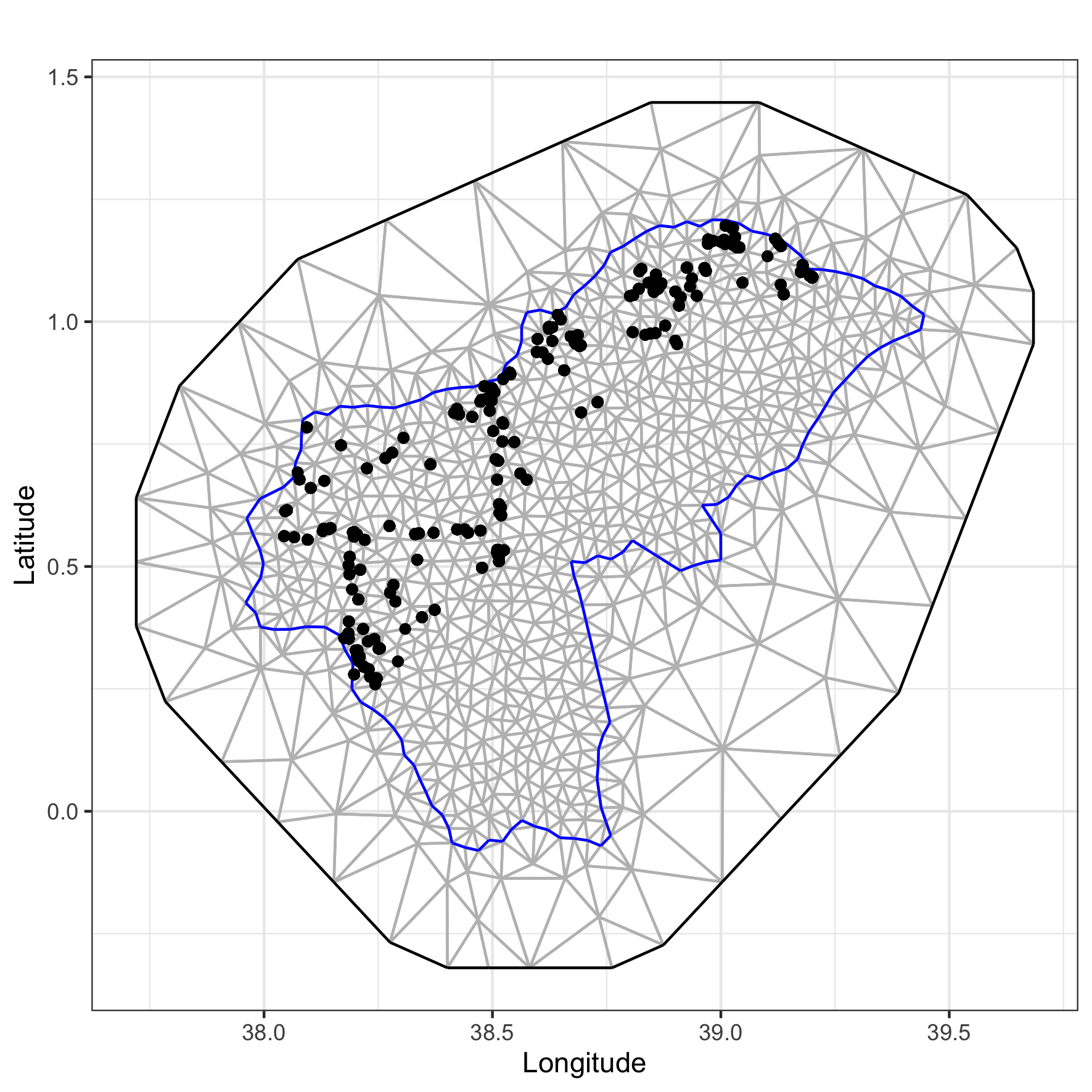


Supplementary figure S1: Discretization of the spatial domain through Delaunay triangulation
